# Supplementary material for: New Conservative Approach for the Management of Recurrent Sublingual Ranula—A Case Report
Source: Int J Environ Res Public Health. 2023 Jan 29;20(3):2398. doi: 10.3390/ijerph20032398 (PMC9915898; doi:10.3390/ijerph20032398)
Supplement: Supplementary file 1 [file ijerph-20-02398-s001.zip › presa d'atto case report new conservative approach for the managemen tof recurrent sublingual ranula.pdf]

Gemelli

Comitato Etico

FONDAZIONE POLICLINICO GEMELLI
PROTOCOLLO UNICO

Tipo Atti: In Uscita
Prot N. 0037733/22 - Del 02/12/2022
.SEGRETERIA COMITATO ETICO

Gent.ma Prof.ssa Patrizia GALLENZI
UOC Odontoiatria generale e ortodonzia

SEDE

Oggetto: Case report "New conservative approach for the management of recurrent sublingual ranula. A case report and brief Review"

Chiarissimo Professore,

si comunica che il Comitato Etico, nella seduta del 10 novembre 2022, ha preso atto dei documenti depositati con email dello 01/11/2022 relativi al case report "New conservative approach for the management of recurrent sublingual ranula. A case report and brief Review.

Il Presidente del Comitato Etico
Prof. Andrea Bacigalupo

Fondazione Policlinico Universitario Agostino Gemelli IRCCS
Università Cattolica del Sacro Cuore
Largo Agostino Gemelli 8, 00168 Roma
T +39 06/30156124 - 5556
comitato.etico@policlinicogemelli.it
www.policlinicogemelli.it

Sede Legale
Largo Francesco Vito 1, 00168 Roma
Sede Operativa
Largo Agostino Gemelli 8, 00168 Roma

Codice Fiscale e Partita IVA 13109681000

Gemelli

ELENCO MEMBRI PRESENTI – SEDUTA DEL 10 NOVEMBRE 2022

Prof. ANDREA BACIGALUPO – Clinico Ematologo
Prof. DON PAOLO BONINI – Esperto di Bioetica
Prof. ALESSANDRO CARUSO – Clinico
Dr. ANTONELLO COCCHIERI – Rappresentante dell'area delle professioni sanitarie
Prof. SEBASTIANO FILETTI – Clinico
Dr. FRANCESCO FILIDORO – Farmacista esperto di dispositivi medici
Avv. DANILO GALLITELLI – Esperto in materia giuridica e assicurativa
Prof.ssa FIORELLA GURRIERI – Esperto di genetica
Dr. MICHELE LEPORE – Medico di Medicina Generale
Dr.ssa GIUSEPPINA LOFFREDI – Rappresentante del volontariato per l'assistenza e/o associazionismo di tutela dei pazienti
Prof. CAMILLO MARRA – Clinico
Dr.ssa BARBARA MEINI - Farmacista
Prof.ssa NADIA MORES – Sostituto permanente del Direttore Sanitario
Prof. GIACOMO POZZOLI – Farmacologo
Prof.ssa KETTY PERIS – Clinico
Prof. DARIO SACCHINI – Esperto di Bioetica
Dr. DOMENICO TARANTINO – Farmacista
